# Development of risk prediction models for preterm delivery in a rural setting in Ethiopia

**DOI:** 10.7189/jogh.13.04051

**Published:** 2023-05-26

**Authors:** Clara Pons-Duran, Bryan Wilder, Bezawit Mesfin Hunegnaw, Sebastien Haneuse, Frederick GB Goddard, Delayehu Bekele, Grace J Chan

**Affiliations:** 1 Department of Epidemiology, Harvard T.H. Chan School of Public Health, Boston, Massachusetts, USA; 2 Machine Learning Department, Carnegie Mellon University, Pittsburgh, Pennsylvania, USA; 3 Department of Pediatrics and Child Health, St. Paul’s Hospital Millennium Medical College, Addis Ababa, Ethiopia; 4 Department of Biostatistics, Harvard T.H. Chan School of Public Health, Boston, Massachusetts, USA; 5 Department of Obstetrics and Gynecology, St. Paul’s Hospital Millennium Medical College, Addis Ababa, Ethiopia; 6 Division of Medical Critical Care, Boston Children’s Hospital, Department of Pediatrics, Harvard Medical School, Boston, Massachusetts, USA.

## Abstract

**Background:**

Preterm birth complications are the leading causes of death among children under five years. However, the inability to accurately identify pregnancies at high risk of preterm delivery is a key practical challenge, especially in resource-constrained settings with limited availability of biomarkers assessment.

**Methods:**

We evaluated whether risk of preterm delivery can be predicted using available data from a pregnancy and birth cohort in Amhara region, Ethiopia. All participants were enrolled in the cohort between December 2018 and March 2020. The study outcome was preterm delivery, defined as any delivery occurring before week 37 of gestation regardless of vital status of the foetus or neonate. A range of sociodemographic, clinical, environmental, and pregnancy-related factors were considered as potential inputs. We used Cox and accelerated failure time models, alongside decision tree ensembles to predict risk of preterm delivery. We estimated model discrimination using the area-under-the-curve (AUC) and simulated the conditional distributions of cervical length (CL) and foetal fibronectin (FFN) to ascertain whether they could improve model performance.

**Results:**

We included 2493 pregnancies; among them, 138 women were censored due to loss-to-follow-up before delivery. Overall, predictive performance of models was poor. The AUC was highest for the tree ensemble classifier (0.60, 95% confidence interval = 0.57-0.63). When models were calibrated so that 90% of women who experienced a preterm delivery were classified as high risk, at least 75% of those classified as high risk did not experience the outcome. The simulation of CL and FFN distributions did not significantly improve models’ performance.

**Conclusions:**

Prediction of preterm delivery remains a major challenge. In resource-limited settings, predicting high-risk deliveries would not only save lives, but also inform resource allocation. It may not be possible to accurately predict risk of preterm delivery without investing in novel technologies to identify genetic factors, immunological biomarkers, or the expression of specific proteins.

Globally, almost 15 million babies are born preterm before 37 weeks of gestation annually [1]. A variety of factors are known to be associated with risk of preterm birth, including obstetrics history, anthropometric measurements, infections, ultrasound measurements, and biological and genetic markers [2,3]. Accurate prediction tools to identify women at an increased risk of preterm delivery would allow policymakers, practitioners, and researchers to target interventions designed to reduce preterm deliveries. Some studies conducted in high income settings developed predictive models to classify women based on their risk for preterm birth, considering multiple maternal characteristics [4,5]. Their discriminative performance was modest (area under the receiving operator characteristic curve (AUC) ranged from 0.62 to 0.70), with generally lower performance when performing external validation [5].

Targeting interventions to high-risk pregnancies is a critical challenge because of the lack of accurate prediction tools. Some published models were developed for women with a priori known risk factors, such as preterm labour or multiple pregnancy [6-8]. Other models used predictors that are not readily available in resource-limited settings, such as cervical length (CL), bacterial vaginosis, foetal fibronectin (FFN), cytokine concentration, and other biomarkers [9-11]. To our knowledge, no published model handled competing risks of stillbirth or considered a combined outcome of preterm delivery regardless of vital status of the foetus or neonate. However, preterm and stillbirth share common causes and risk factors, and it is likely that the biological mechanisms that trigger preterm labour or rupture of membranes may lead to the delivery of a preterm stillborn in extreme cases [12,13]. Overall, there is a gap in the development of prediction tools that are accurate and applicable to the general population, with and without a priori risk, especially in low-resource countries where data on biomarkers that could contribute to improve model performance are not commonly available.

Most preterm birth cases occur among women without known risk factors [14,15]. Limited availability of promising biomarkers to predict preterm birth in low-resource settings make it critical to develop context-specific predictive tools. We used large data sets from the Birhan pregnancy cohort [16] to test the possibility of predicting risk of preterm delivery in rural Ethiopia and to ascertain whether it would be effective to invest in the collection of known key predictors such as CL or FFN to improve accuracy of predictions by simulating their conditional distribution.

## METHODS

### Study design and setting

We conducted a cohort study in the Birhan field site, including 16 villages in Amhara region, Ethiopia, covering a population of 77 766, to estimate morbidity and mortality outcomes among 17 108 women of reproductive age and 8554 children under-five with trimonthly house-to-house surveillance. The site, established in 2018, is a platform for community and facility-based research and training, focused on maternal and child health [17]. Nested in it is an open pregnancy and birth cohort that enrols approximately 2000 pregnant women and their newborns annually, with rigorous longitudinal follow-up over the first two years of life and household data linked with health facility information [16]. The catchment area is rural and semi-urban, covering both highland and lowland areas and including two different districts, Angolela Tera, and Kewet/Shewa Robit.

We used data from the Birhan Health and Demographic Surveillance System (HDSS) and the nested pregnancy and birth cohort (Birhan Maternal and Child Health (MCH)) to develop a series of risk prediction models for preterm delivery [16,17]. The HDSS provides estimates and trends of health and demographic outcomes, including morbidity among women of reproductive age and children under two years and births, deaths, marriages, and migration in the entire population [17]. The pregnancy and birth cohort generates evidence on pregnancy, birth, and child outcomes using clinical and epidemiological data at both the community and health facility level [16].

### Study participants

The study sample included women enrolled during pregnancy in the MCH cohort between December 2018 and March 2020, followed-up in home and facility visits through delivery beyond 28 weeks of gestation. This gestational age cut-off was used because stillbirths in Ethiopia are considered ≥28 weeks. We excluded newborns with implausible gestational ages at birth: <28 weeks due to the definition of stillbirth, and ≥46 weeks.

### Study variables and definitions

The study outcome was preterm delivery, a composite indicator defined as any delivery occurring before 37 completed weeks of gestation, regardless of vital status of the foetus or neonate. This included both preterm births (live birth prior to completion of week 37 of gestation) [18] and stillbirths (any foetal death after 28 completed weeks of gestation) [19] which occurred before 37 weeks of gestation.

Gestational age was estimated using the best available method from ultrasound measurements, reported date of last menstrual period, fundal height, or maternal recall of gestational age in months. Detailed information on these estimations can be found elsewhere [20].

The selection of potential predictors was guided by literature review and expert knowledge from study obstetricians and paediatricians. Predictors with low prevalence rates in the sample (rare events with ≤5 cases) were dropped. We included over 70 socio-demographic, biological, environmental and pregnancy-related predictors in the initial models. The complete list of assessed predictors can be found in Table S1 in the **Online Supplementary Document**. We included dummy variables indicating missingness for each predictor as additional variables, an approach justified for predictive models because it reflects the complete state of knowledge available at the time of prediction.

### Analysis

#### Descriptive statistics

We performed a descriptive analysis of the background characteristics of women who experienced term compared to preterm delivery using *t*-test for continuous variables, χ^2^ test for most of the binary variables, and Fisher exact test for multiple gestations, to test for statistically significant differences between groups.

#### Prediction models

We fit five models to predict risk of preterm delivery, including linear models and nonlinear decision tree approaches. All five strategies were designed to predict the outcome of preterm delivery using information available at 28 weeks of gestation. The first four models used time-to-event methods, which modelled the time until delivery from the 28^th^ week gestation mark, accounting for left truncation and right censoring of person-time. Left truncation arises when women are enrolled beyond 28 weeks of gestation while right censoring arises when follow-up ceases prior to observation of the event of interest (e.g. due to outmigration or loss-to-follow-up). First, we fit a Cox proportional hazards model using the R package survival [21]. Second, we fit accelerated failure time model which we fit with a log-logistic distribution using the R package flexsurv [22]. Third, we fit a decision tree using the R package LTRtrees that extends previous uses of a decision tree in survival analysis to account for left truncation and right censoring (left truncation right censoring classification and regression trees (LTCART)) [23]. Fourth, we implemented a decision tree ensemble using the eXtreme Gradient Boosting (XGB) R package which uses a Poisson likelihood function proposed by Fu and Simonoff (2017) to account for right censoring and left truncation [23,24]. Finally, we fit a fifth analysis based on a XGB classification model using a binary outcome (i.e. whether delivery was preterm or not) instead of the time-to-event; during the fitting of this model, we excluded data which was either right censored or left truncated.

Models were fit and evaluated using 5-fold cross validation due to the need to evaluate models on out-of-sample data while reserving as much data as possible for fitting [25]. All models were evaluated on the same held-out data set within each fold, regardless of which data or methods were used while fitting the model. Model performance was assessed using the AUC to assess accuracy at binary classification, and the c-index to assess the fraction of pairs for which predicted risk was concordant with delivery time. For both metrics, a value of 0.5 represents a random prediction which is uncorrelated with the true outcome. Larger values indicate more accurate predictions, and a value of 1 represents predictions which are perfectly concordant with the true outcome.

#### Simulation of cervical length and foetal fibronectin

We performed a final analysis to simulate the potential impact of including CL and FFN as predictors. These two variables were found in past work to be significantly associated with preterm birth [26-28]. Since they are not regularly collected in the study region, we used simulation to assess the potential gain from collecting them. The simulation used data from the MFMU PREDS study [29], a study which screened 2929 women for risk factors for preterm birth in the United States. PREDS study identified CL and FFN as key predictors for preterm birth [30,31]. Details on the simulation model and comparison between the simulated and real measurements can be found in the Supplemental Methods and Results in the **Online Supplementary Document**.

## RESULTS

The sample composed 2834 pregnancies. We excluded 75 (2.6%) records with gestational age at delivery <28 and ≥46 weeks and 266 (9.4%) pregnancies whose follow-up did not go beyond 28^th^ gestational week. We included 2493 pregnancies in the study; a further 138 (5.5%) women were lost to follow-up before delivery or did not have a recorded gestational age at delivery, so we treated them as censored observations in the time-to-event models and excluded from the binary classification model. We enrolled 968 (38.8%) women in the cohort after 28 weeks of gestation (left-truncation), so we considered their time-varying predictors as missing since no information on those factors was available at the time of prediction; we also excluded them from the binary classification model.

Among the 2355 women included in the study and followed until delivery, 14% had a preterm delivery. There was no difference in some background characteristics like age, body mass index, parity, or history of previous preterm births among women with term deliveries compared to women with preterm deliveries (**Table 1**). However, the two groups differed significantly in literacy (43.3% of women with term delivery were illiterate, compared to 50.5% of those who delivered prematurely), geographic location (42.8% of term deliveries occurred in the highland district within Birhan field site, compared to 52.4% of preterm deliveries), and multiple gestation (1.1% of term deliveries were multiple, compared to 3.4% of preterm deliveries).

**Table 1:** Characteristics of the study sample

Variables	Total*			Term delivery			Preterm delivery			
	**N**	**mean**	**SD**	**N**	**mean**	**SD**	**N**	**mean**	**SD**	***P*-value**
Maternal age (years)	2487	27.3	6.1	2030	27.3	6	319	27.2	6.4	0.69
Body mass index (preconception)	1108	22.0	3.1	930	22.0	3.2	138	21.9	2.6	0.60
	**N**	**n**	**%**	**N**	**n**	**%**	**N**	**n**	**%**	***P*-value**
Illiterate	2488	1105	44.4	2031	880	43.3	319	161	50.5	0.02
Location: highland district (Angolela Tera)	2489	1075	43.2	2032	870	42.8	319	167	52.4	<0.01
Primiparous	2493	793	31.8	2034	627	30.8	321	108	33.6	0.34
History of a previous preterm birth	2493	47	1.9	2034	38	1.9	321	7	2.2	0.87
Multiple gestation	2408	35	1.5	2034	23	1.1	321	11	3.4	<0.01
Neonatal sex: female	2355	1144	48.6	2034	993	48.8	321	151	47.0	0.59

SD – standard deviation

*Total counts include censored study participants with unknown pregnancy outcome or date of delivery.

Some predictors had high levels of missing data, particularly where our study relied on facility visits. Approximately 25% of participants did not attend any antenatal care visit in the study health facilities after being enrolled in the cohort; thus, variables collected at antenatal care visits, such as current infections or concomitant diseases, were missing for these women. Further, over 70% of women who attended at least one antenatal care visit had missing data on laboratory and point-of-care results such as white blood cell counts, proteinuria or bacteriuria.

**Figure 1:**
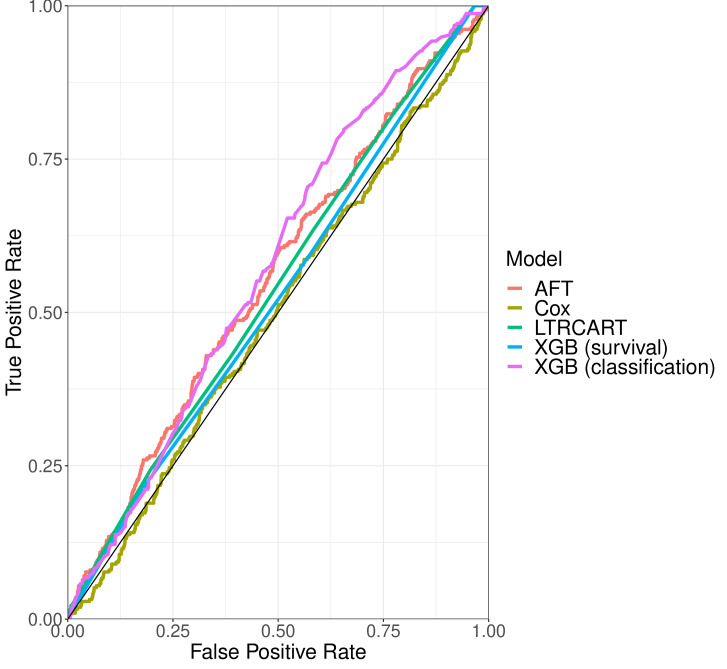
ROC curves for each model. AFT – accelerated failure time, LTRCART – left truncation right censoring classification and regression trees, ROC – receiver operating characteristic curve, XGB – eXtreme gradient boosting.

The predictive performance of all models was generally poor (**Table 2**). The c-statistic and AUC were highest for the XGB classification model (AUC = 0.60; 95% confidence interval (CI) = 0.57-0.63). The receiver operating characteristic curves (ROC) depict the trade-off between the false and true positive rates achieved by varying the threshold for classifying delivery as preterm or term (**Figure 1**). As an example, at the point on this curve corresponding to a 90% true positive rate, all models had a false positive rate of at least 75%, indicating a lack of specificity in picking out women who are truly at higher risk.

**Table 2:** Performance metrics of the different predictive models

Model	AUC (95% CI)	c-statistic (95% CI)
Accelerated failure time	0.57 (0.54-0.61)	0.53 (0.52-0.55)
Cox	0.51 (0.47-0.54)	0.52 (0.50-0.53)
LTRCART	0.54 (0.51-0.58)	0.53 (0.51-0.55)
XGBoost (classification)	0.60 (0.57-0.63)	0.54 (0.52-0.55)
XGBoost (survival)	0.52 (0.49-0.56)	0.52 (0.50-0.53)

AUC – area under the receiver operating characteristic curve, CI – confidence interval, LTRCART – left truncation right censoring classification and regression trees, XGBoost – eXtreme gradient boosting

There was substantial heterogeneity in the factors that were ultimately retained in the five models (**Table 3**). Both biological and socio-demographic factors were among the top contributors of standard time-to-event models. Regarding decision tree models, the top five predictors are mainly biological, with neonatal sex being the predictor with the greatest importance.

**Table 3:** Performance metrics of the different predictive models

Model	Top predictors				
	**1**	**2**	**3**	**4**	**5**
**Accelerated failure time**	Multiple gestation	Lymphocyte % = NA	Vaginal bleeding	History of pre-eclampsia	Maternal age = NA
Accelerated factor	0.74	1.26	1.22	0.83	0.83
**Cox**	Multiple gestation	History of diabetes	Maternal age = NA	Lymphocyte % = NA	History of pre-eclampsia
Hazard ratio (95% CI)	3.15 (0.61-16.21)	0.33 (0.03-3.61)	2.79 (1.72-4.55)	2.68 (0.10-1.63)	2.29 (1.44-3.62)
**LTRCART**	Neonatal sex	Multiple gestation	NA	NA	NA
Split level	1	2	-	-	-
**XGBoost (classification)**	Location (district)	Parity	Family size = top quintile	Neonatal sex	Systolic blood pressure
Gain	0.11	0.10	0.10	0.09	0.08
**XGBoost (survival)**	Neonatal sex	NA	NA	NA	NA
Gain	1.00	-	-	-	-

LTRCART – left truncation right censoring classification and regression trees, NA – not applicable/missing data, CI – confidence interval, XGBoost – eXtreme gradient boosting

The performance of several individual models improved when simulated measurements of CL and FFN were included as features for each individual, particularly the accelerated failure time and LTRCART decision tree models (**Table 4**). However, no model exceeded an estimated AUC of 0.60, indicating that the overall predictability of preterm delivery did not change substantially from the inclusion of these additional predictors.

**Table 4:** Performance metrics of the models with simulated measurements

Model	AUC (95% CI)	c-statistic (95% CI)
Accelerated failure time	0.60 (0.56-0.63)	0.55 (0.53-0.57)
Cox	0.55 (0.52-0.59)	0.53 (0.50-0.55)
LTRCART	0.60 (0.56-0.64)	0.55 (0.52-0.57)
XGBoost (classification)	0.60 (0.56-0.64)	0.55 (0.52-0.56)
XGBoost (survival)	0.48 (0.45-0.51)	0.50 (0.48-0.51)

AUC – area under the receiver operating characteristic curve, CI – confidence interval, LTRCART – left truncation right censoring classification and regression trees, XGBoost – eXtreme gradient boosting

## DISCUSSION

Our study shows that risk prediction of preterm delivery remains a challenge in the absence of data on biomarkers. Despite using a wide range of methodological approaches to adjust for missing data, competing risk of stillbirths, and late cohort enrolments, both traditional epidemiological and machine learning models performed poorly and had low specificity in identifying women who delivered before 37 weeks of gestation. This low predictive performance differs from existing models with higher predictive ability that were designed to predict preterm birth among women at an already high risk due to obstetric conditions such as twin pregnancy [7,8], short cervix, cervical insufficiency [10,32], or hospital admission due to preterm labour [6,33,34]. Other higher performing algorithms used predictors that were not available or applicable in low-resource settings such as amniotic and cervical fluids [33], inflammatory markers [35], or method of conception [5,8]. Lack of a fixed prediction time point and competing risk of stillbirth are methodological gaps of most published studies.

To our knowledge, our study is among the few in developing risk prediction models in a low-resource setting. Only one published study presented the development of a model for preterm birth prediction in Ethiopia [36]. Despite reporting good model performance, this study has an important limitation; it predicts preterm birth retrospectively using all information available in hindsight (e.g. events such as premature rupture of membranes), while our aim is to assess whether early prediction is possible to inform preventive interventions, leading us to fix a time point for prediction (28 weeks of gestation). Moreover, their study was conducted using data from a hospital-based cohort, likely to be composed of women at higher a priori risk of adverse outcomes.

Studies carried out in Ethiopia identified risk factors for preterm delivery including obstetric conditions, socio-demographic characteristics, urinary and vaginal infections, and hypertensive disorders [37,38]. We included all these factors to build the most accurate models with the available data. Among all predictors, neonatal sex was assigned high importance in the decision tree models despite the small difference in prevalence between boys and girls. This is consistent with higher rates of preterm birth for male foetuses in other studies [39,40]. Nevertheless, prediction studies like this aim to characterise prognosis and to anticipate or forecast an outcome.

We simulated the conditional distribution of CL and FFN to test whether access to these measurements may improve models’ predictive ability. However, the improvement of the models was negligible, indicating that women identified as high-risk via CL or FFN could also be identified as high risk via other predictors. Although both CL and FFN are among the most used indicators to identify high risk pregnancies for preterm delivery in clinical practice, their measurement is not always recommended in a priori low-risk populations [15,41]. Our results do not support the allocation of resources to CL and FFN measurement in low-resource settings to predict risk of preterm birth. Similarly, other studies observed a poor predictive power of CL and FFN in the absence of additional maternal predictors [42].

Our findings have important research-related and public health implications. Given the poor performance of all available predictive models, research on the underlying causes of preterm delivery must be continued to achieve a better understanding of the pathways between different risk factors and preterm birth in order to predict and prevent future preterm births. Most cases still occur among women without any known risk factor [14,15]. It is crucial to look for new indicators and biomarkers of preterm delivery. Genetic factors, immunological biomarkers, and protein expression are showing promising results [43,44]. There may be value in exploring the use of “omics”, since no biomarkers predictive of preterm birth have yet been identified [45]. While all settings can benefit from such technologies, from an equity perspective it may be especially important to ensure availability in low-resource settings where the survival of preterm infants is lower and identifying high-risk women can enable targeted preventative interventions.

Predictive algorithms with modest performance could be used to identify pregnancies at a very low risk of preterm delivery, thus excluding them from interventions. However, a large proportion of women at low risk will still be targeted in those interventions due to the models’ low specificity. In Ethiopia, recommending pregnant women to stay in maternity waiting homes is part of the birth preparedness strategy, though it has not been demonstrated to improve pregnancy outcomes [46,47]. Targeting the recommendation of staying in maternity waiting homes to a reduced number of individuals would increase the cost-effectiveness of the intervention and improve the pregnancy experience of some women.

Our findings should be interpreted considering some limitations. Like most longitudinal studies, we had study attrition. To address loss to follow-up, we adjusted time-to-event models for censoring and created a “missing” category for all predictors with missing data. The use of a composite outcome that included all preterm deliveries regardless of vital status of the foetus or neonate did not enable the models to separately predict the risk of having a live preterm baby from the risk of having a preterm stillbirth. However, the use of a combined outcome allows us to address competing risks of preterm stillbirths, a common limitation of other available prediction models. Despite these limitations, our study fills an evidence gap by exploring prediction of preterm delivery during the first 28 weeks of gestation in a resource-limited setting with important restrictions in data availability. We considered a comprehensive selection of >70 predictors and tested five different algorithms: Accelerated Failure Time, Cox, LTRCART, and two decision tree ensembles. We acknowledge that the classification tree ensemble is not recommended for data with censoring or truncation. However, we fit this model together with the other four algorithms for the purpose of being fully exhaustive in our effort to explore all potential methodological options to achieve our study aim of developing an accurate algorithm. Their results shows that the difficulty of predicting preterm delivery is robust to potential variation in the process of constructing risk models.

## CONCLUSIONS

In settings with low coverage of antenatal care and limited resources to perform ultrasound and biomarker measurements, predicting risk of preterm delivery remains a major challenge. New indicators of preterm delivery may be necessary to enable targeted interventions.

## Additional material


Online Supplementary Document
