# Supplementary material for: Development of risk prediction models for preterm delivery in a rural setting in Ethiopia
Source: J Glob Health. 2023 May 26;13:04051. doi: 10.7189/jogh.13.04051 (PMC10208651; doi:10.7189/jogh.13.04051)
Supplement: Online Supplementary Document [file jogh-13-04051-s001.pdf]

ONLINE SUPPLEMENTARY DOCUMENT

Development of risk prediction models for preterm delivery in a rural setting in Ethiopia

Table of contents

Table S1. Predictors assessed for inclusion in the models, pages 2-11

Supplemental Methods and Results. Simulation of cervical length and fetal fibronectin: methods and fit between simulated and real values, pages 12-13

Table S2. Coefficients for simulated model of cervical length and fetal fibronectin, page 12

Figure S1. Joint distribution of gestational age and cervical length in the PREDS dataset (left) and simulated values (right). The blue line gives a LOWESS smoothing, page 13

Figure S2. Joint distribution of fetal fibronectin (1: positive, 0: negative) and cervical length in the PREDS dataset (left) and simulated values (right). The blue line gives a LOWESS smoothing, page 13

Table S1. Predictors assessed for inclusion in the models

Predictors	Included in the models	Reasons for exclusion	Other remarks
**Socioeconomic and demographic**			
Maternal age	Yes		
Maternal education	Yes		
Maternal literacy	Yes		
Marital status	Yes		
Family has own income	Yes		
Main source of income	Yes		
Wealth index	Yes		
Family size (number of individuals in the household)	Yes		
Ethnicity	Yes		
Religion	Yes		
Urban/rural area	Yes		Woreda used as a proxy
Altitude	Yes		Woreda used as a proxy
Time and distance to the nearest health facility	Yes		
**Environmental and behavioral**			
Household air pollution	No	Not available in the study database	
Alcohol intake	Yes		
Tobacco use	No	No smokers in the sample	

Table S1. Predictors assessed for inclusion in the models (continued)

Substance abuse	No	Rare event (≤5 cases)	
Iron intake	Yes		
Folic acid intake	Yes		
Mebendazole intake	Yes		
Other medication intake	Yes		
Multivitamin use	Yes		
Vitamin A intake	Yes		Intake of Vitamin A rich fruits and vegetables
Zinc intake	No	Not available in the study database	
Oral rehydration salts	No	Not available in the study database	
Nutrition: intake of different products (fruits, vegetables, oil, coffee, meet, eggs, fish)	Yes		
Fasting during pregnancy	Yes		
Insecticide-treated nets use	Yes		
**Anthropometric measurements**			
Height	Yes		Preconception measurement
Weight	Yes		Preconception measurement
Body Mass Index	Yes		Preconception measurement
Mid upper arm circumference	Yes		Measurement from enrolment visit used as a proxy of preconception measurement

Table S1. Predictors assessed for inclusion in the models (continued)

**History of previous diseases and conditions**			
Diabetes	Yes		
Eclampsia	Yes		Combined with pre-eclampsia
Pre-eclampsia	Yes		Combined with eclampsia
Sexually transmitted diseases	Yes		
Cardiac disease	No	Rare event (≤5 cases)	
Chronic hypertension	No	Rare event (≤5 cases)	
Renal disease	No	Rare event (≤5 cases)	
Severe medical disease or condition	No	Rare event (≤5 cases)	
**Obstetric history**			
Previous multiple gestation	Yes		
Previous stillbirth	Yes		
Previous miscarriage	Yes		
Previous preterm delivery	Yes		
Previous low birth weight neonate	Yes		
Previous newborn with birth defect	No	Rare event (≤5 cases)	
Previous caesarean section	Yes		
Previous antepartum hemorrhage	No	Not available in the study database	

Table S1. Predictors assessed for inclusion in the models (continued)

Interpregnancy interval	Yes		
Parity	Yes		
Gravidity	Yes		
History of surgeries in reproductive tract	Yes		
Method of conception	No	Not available in the study database	
**Current infections and concomitant diseases**			
Amniotic fluid infection	No	Not available in the study database	
Bacterial vaginosis	No	Not available in the study database	
Chlamydia	No	Not available in the study database	
Maternal chorioamnionitis	No	Rare event (≤5 cases)	
Syphilis	Yes		
Pelvic infection	No	Not available in the study database	
Malaria	No	Not available in the study database	
HIV	Yes		
Hepatitis B	No	Rare event (≤5 cases)	
Sickle cell anemia	No	Not available in the study database	
Eclampsia	No	Rare event (≤5 cases)	

Table S1. Predictors assessed for inclusion in the models (continued)

Pre-eclampsia	No	No cases of pre-eclampsia in the sample	
Asthma	No	Not available in the study database	
Diabetes	No	Only 2 women screened for diabetes	
Mental disorder	No	Not available at origin (week 28 of gestation)	
Thyroid disease	No	Not available in the study database	
Pregnancy induced hypertension	No	Rare event (≤5 cases)	
Psychological and social stress	No	Not available in the study database	
Chest abnormality	No	Rare event (≤5 cases)	
Heart abnormality	No	Rare event (≤5 cases)	
Depression	No	Not available at origin (week 28 of gestation)	
**Antenatal care**			
Number of antenatal care visits	Yes		
Early antenatal care attendance	Yes		Two predictors considered: first ANC visit before week 13 and before week 24

Table S1. Predictors assessed for inclusion in the models (continued)

**Biological, signs and symptoms**			
Multiple gestation	Yes		Information collected at delivery. Suspected multiple gestation for women who were lost to follow-up
Neonatal sex	Yes		Information collected at delivery. Unknown for women who were lost to follow-up
Preterm labor	No	Rare event (≤5 cases)	
Premature rupture of membranes	No	Rare event (≤5 cases)	
Vaginal bleeding	Yes		
Antepartum hemorrhage	No	Rare event (≤5 cases)	
Vaginal discharge syndrome	No	Rare event (≤5 cases)	
Genital ulcer	No	Rare event (≤5 cases)	
Pallor	Yes		
Jaundice	Yes		
Severe nausea and vomiting	Yes		
Headache	Yes		
Blurred vision	Yes		
Right upper quadrant abdominal pain	Yes		
Fever	Yes		
Pain during urination	Yes		

Table S1. Predictors assessed for inclusion in the models (continued)

Increased urinary frequency	Yes		
Increased urinary urgency	Yes		
Constipation	No	Rare event (≤5 cases)	
Painful sexual intercourse	No	Not available in the study database	
Seizure	No	Not available in the study database	
Fetal movement recall	Yes		
Composite predictor: Danger signs and symptoms	Yes		At least one of the following signs and symptoms: headache, blurring of vision, vaginal bleeding, urinary pain, urinary frequency, urinary urgency, decreased foetal movement, vaginal discharge syndrome, right upper quadrant abdominal pain, fever, pallor, jaundice, severe vomiting or nausea
Composite predictor: Severe maternal complications	No	Rare event (≤5 cases)	At least one of the following maternal complications: premature rupture of membranes, antepartum haemorrhage and pregnancy induced hypertension
**Ultrasound measurements**			
Cervical length (CL)	No	Not available in the study database	

Table S1. Predictors assessed for inclusion in the models (continued)

Cervical glandular area	No	Not available in the study database	
Foetal breathing movements	No	Not available in the study database	
Biometry	No	Not available in the study database	
Intrauterine growth restriction	No	Not available in the study database	
**Other laboratory, non-laboratory, and point-of-care measurements**			
Foetal heartbeat	No	Not available at origin (week 28 of gestation)	
Foetal respiratory rate	No	Not available in the study database	
0_2 saturation	No	Not available in the study database	
Blood pressure	Yes		
Hb / anaemia	Yes		
Haematocrit	Yes		
Blood glucose levels	No	Only 2 women with measured blood glucose levels	
Blood group	Yes		
Rh factor	Yes		
White blood cells (in x1000 cells/µl)	Yes		
Neutrophils (%)	Yes		

Table S1. Predictors assessed for inclusion in the models (continued)

Lymphocytes (%)	Yes		
Nitrites in urine	No	Rare event (≤5 cases)	
Leukocyte esterase in urine	Yes		
Bacteriuria	Yes		
Proteinuria	Yes		
Micronutrient levels in blood	No	Not available in the study database	
Hormone levels (cortisol, corticotropin-releasing hormone, salivary estriol, human chorionic gonadotropin, relaxin)	No	Not available in the study database	
Fetal Fibronectin (FFN)	No	Not available in the study database	
Alpha fetoprotein level	No	Not available in the study database	
Pregnancy-associated plasma protein A (PAPP-A)	No	Not available in the study database	
Interleukin levels	No	Not available in the study database	
Vaginal microbiota	No	Not available in the study database	
Other cervical, amniotic fluid and blood biomarkers (alkaline phosphatase, β-2-microglobulin, C-reactive protein, defensins, IGFBP-1, matrix metalloproteinase-8, granulocyte colony stimulating factor)	No	Not available in the study database	

Table S1. Predictors assessed for inclusion in the models (continued)

Genetic polymorphisms	No	Not available in the study database	

Note: all predictors included in the models were generated with information available at origin/time of prediction (week 28 of gestation), or using data collected later in pregnancy only if the predictor was time-invariant.

Supplemental Methods and Results. Simulation of cervical length and fetal fibronectin: methods and fit between simulated and real values

The simulation modelled the joint distribution of cervical length (CL) and fetal fibronectin (FFN) conditioned on the true gestational age of the delivery, to capture the fact that both variables contain (potentially noisy) information about the outcome of interest. We modelled CL as a continuous variable and FFN as a binary variable (i.e., a positive or negative test result). Their joint distribution was also conditioned on age, parity, and body mass index (BMI), three variables associated with CL and FFN which are present in both datasets. This helped adjust the simulation model to account for differences between the two populations. A factorization of the joint distribution was specified as

$$Pr[CL, FFN] = Pr[CL] \cdot Pr[FFN|CL] = N\left(\beta_{CL,\mu}x, \beta_{CL,\sigma}x\right) \cdot \ \sigma(\beta_{FFN}[\chi\ CL])$$

where N is the normal distribution, σ is the inverse logit function and the β are parameters which control the mean and variance of each term. The model was fit using maximum likelihood on the PREDS dataset. Table S2 shows the fitted values for each coefficient. Measurements for CL and FFN were simulated from the model for our MCH cohort using these inferred parameters. To assess the fidelity of the simulation, we also simulated measurements for the PREDS cohort and compared the joint distribution of gestational age and each measurement in the PREDS dataset versus simulation. Figure S1 shows the real and simulated joint distribution of CL and gestational age. Figure S2 shows the real and simulated joint distribution of FFN and gestational age. We observe a high degree of consistency between the real and simulated joint distributions for both measurements, though some discrepancies are present in the conditional mean of the right tail of the FFN-gestational age distribution. This is due to the very small number of data points which fall into this part of the distribution.

Table S2. Coefficients for simulated model of cervical length and foetal fibronectin.

Variable	$\boldsymbol{\beta_{CL,\mu}}$	$\boldsymbol{\beta_{CL,\sigma}}$	$\boldsymbol{\beta_{FFN}}$
Gestational age (weeks)	0.629	-0.247	-0.211
Age (years)	0.057	0.051	0.007
Parity	2.000	0.315	0.306
BMI	0.088	0.039	0.020
Cervical length (mm)	N/A	N/A	-0.058
Intercept	6.036	15.087	5.461

BMI – Body Mass Index; CL – Cervical Length; FFN – Foetal Fibronectin

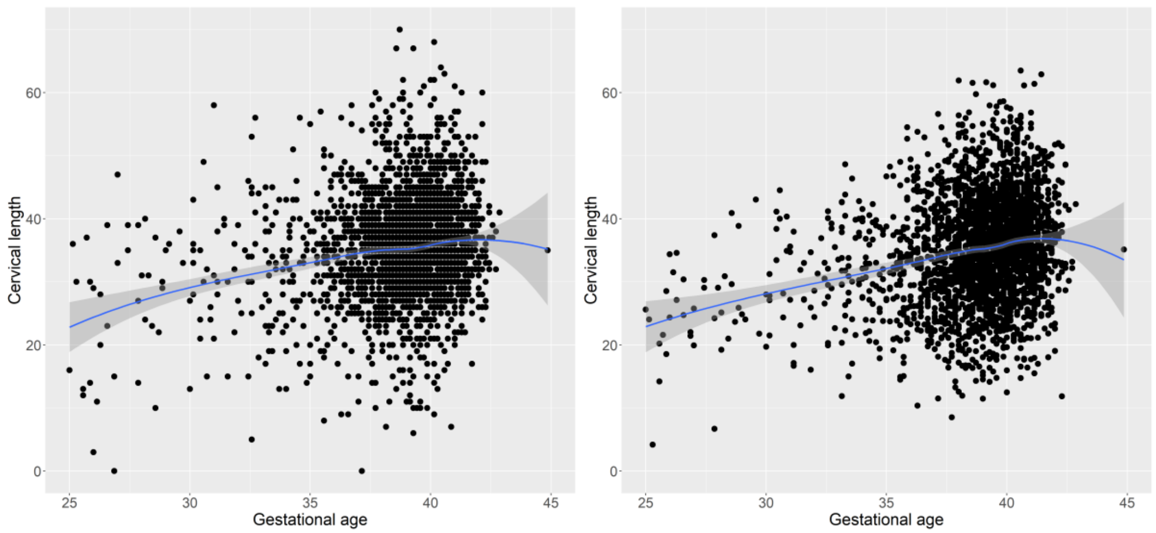

Figure S1. Joint distribution of gestational age and cervical length in the PREDS dataset (left) and simulated values (right). The blue line gives a LOWESS smoothing.

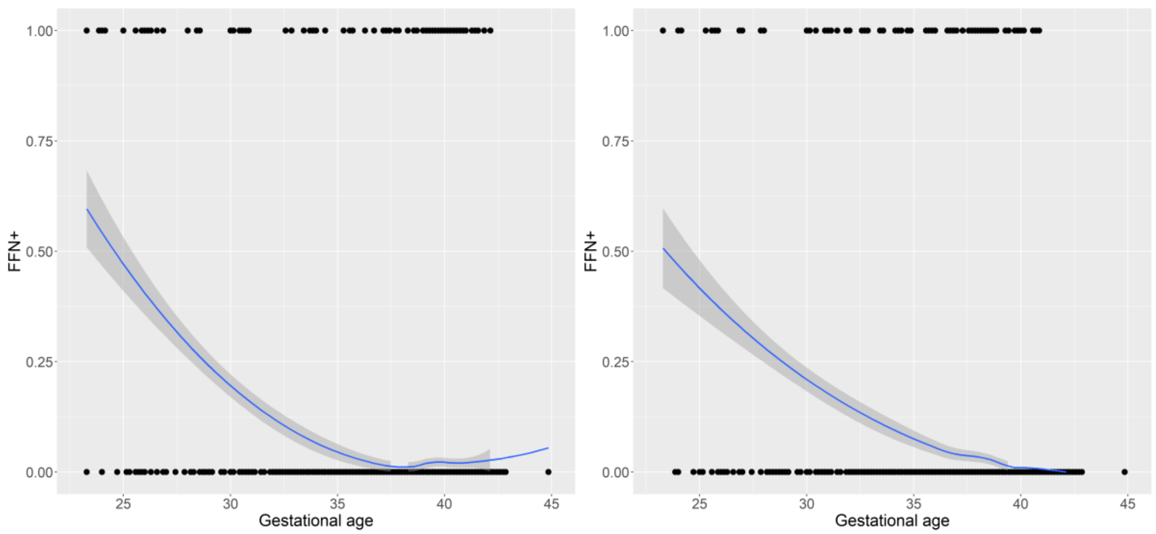

Figure S2. Joint distribution of fetal fibronectin (1: positive, 0: negative) and cervical length in the PREDS dataset (left) and simulated values (right). The blue line gives a LOWESS smoothing.
